# Allosteric and ATP-Pocket BCR::ABL1 Inhibition In Vitro, and Characterising Ex Vivo Thrombo-Inflammatory Biomarkers and Thrombin Generation in Asciminib-Treated CML Patients

**DOI:** 10.3390/ijms27083623

**Published:** 2026-04-18

**Authors:** Musab M. A. Omar, Majed A. Alanazi, David T. Yeung, Timothy P. Hughes, Denise E. Jackson

**Affiliations:** 1 Thrombosis and Vascular Diseases Laboratory, School of Health and Biomedical Sciences, STEM College, RMIT University, Melbourne, VIC 3000, Australia; s3614052@student.rmit.edu.au (M.M.A.O.);; 2 Department of Clinical Laboratory Sciences, College of Applied Medical Sciences, Taibah University, Madinah 42353, Saudi Arabia; 3 Ministry of Education, Riyadh 12435, Saudi Arabia; 4 Haematology, SA Pathology, Adelaide, SA 5000, Australia; 5 Precision Cancer Medicine Theme, South Australian Health & Medical Research Institute, Adelaide, SA 5000, Australia; tim.hughes@sahmri.com; 6 School of Medicine, University of Adelaide, Adelaide, SA 5005, Australia

**Keywords:** CML, platelets, haemostasis, thrombosis, inflammation

## Abstract

Chronic myeloid leukaemia (CML) is driven by the t(9;22) forming the BCR::ABL1 fusion gene, leading to the development of hyper-myeloid proliferation. This led to development of tyrosine kinase inhibitors (TKIs) such as Imatinib, Nilotinib, and Ponatinib. However, resistance or intolerance to ATP-competitive TKIs remains a challenge for some patients. asciminib (ABL001), a novel TKI, targets the myristoyl pocket of ABL1 instead of the ATP-binding site, reducing resistance to mutations. As asciminib is linked to thrombocytopenia, its effects on platelet activation, endothelial function, and inflammation must be studied to assess its potential to promote thrombosis. The main objective of this study is to determine the potential of asciminib as a monotherapy in inducing pathological responses to platelets and endothelium over time within the vasculature. This study assessed the effects of TKIs including asciminib on platelets and thrombotic biomarkers. Washed platelets were used to measure granule secretion, thrombus formation, surface expression of glycoproteins, apoptosis, and viability. Plasma from chronically Asciminib-treated CML patients was analysed using sandwich ELISA for inflammatory and platelet–endothelial biomarkers, and thrombin generation assays were performed to study coagulation. This approach combined in vitro and ex vivo methods to explore the impact of asciminib on platelet function and thrombotic potential. The study shows that acute treatment with asciminib does not promote platelet activation or thrombus formation. Instead, it exhibits an inhibitory effect on thrombus formation in vitro and is associated with reduced thrombo-inflammatory biomarkers ex vivo in chronically treated CML patients. Asciminib was associated with increased thrombin generation over time, suggesting an effect on secondary haemostasis. Asciminib does not appear to induce a prothrombotic or proinflammatory state under the conditions studied, which may be advantageous for CML patients. However, the observed increase in thrombin generation over time suggests a potential effect on secondary haemostasis that warrants further investigation in controlled studies.

## 1. Introduction

Tyrosine kinase inhibitors (TKIs) are targeted therapies used in the treatment of chronic myelogenous leukaemia (CML) and Philadelphia chromosome-positive acute lymphoblastic leukaemia (ALL) [1]. CML is defined by the presence of the Philadelphia chromosome, arising from a reciprocal translocation between chromosomes 9 and 22 that generates the BCR::ABL1 fusion gene in haematopoietic progenitor cells, resulting in constitutive tyrosine kinase activity that drives uncontrolled myeloid proliferation and disease progression [1]. ATP-competitive TKIs, including Imatinib, Nilotinib, and Ponatinib, inhibit BCR::ABL1 by binding to its ATP-binding site, thereby suppressing oncogenic signalling and pathological myeloid proliferation [1]. The overarching goals of CML management include maintaining quality of life, minimising treatment-related toxicity, preventing progression to accelerated or blast phase, and achieving durable haematological and cytogenetic remission to ensure long-term survival [2]. Asciminib, previously referred to as ABL001, was approved by the Food and Drug Administration (FDA) in October 2021 as a first-in-class allosteric myristoyl inhibitor and a third-line treatment for CML [3].

Asciminib has demonstrated a favourable safety profile and clinically meaningful superior efficacy compared with ATP-competitive inhibitors such as Bosutinib [4]. Mechanistically, asciminib is distinct from ATP-competitive TKIs as it targets the ABL1 myristoyl pocket (STAMP). In native ABL1, autoinhibition is mediated by binding of the N-terminal myristoylated residue encoded by exon 1 to this distal pocket; this regulatory mechanism is lost in BCR::ABL1 due to fusion-associated deletion of ABL1 exon 1. Asciminib functionally mimics the missing myristoyl interaction, restoring the inactive conformation of ABL1 and suppressing kinase activity [5].

ATP-competitive TKIs, particularly Nilotinib and Ponatinib, are effective in CML but are associated with increased rates of cardiovascular disease, including arterial occlusive events. These adverse outcomes have been linked, at least in part, to platelet activation, aggregation, and thromboinflammatory mechanisms, with additional evidence implicating endothelial dysfunction, necessitating close clinical monitoring [6,7,8,9,10]. In this context, asciminib represents a mechanistically distinct therapeutic strategy, although continued vigilance and longer-term follow-up remain necessary to fully characterise its cardiovascular risk profile.

In the present study, we investigated the effects of asciminib in comparison with ATP-pocket BCR::ABL1 inhibitors (Imatinib, Nilotinib, and ponatinib) on platelet function using healthy human platelet suspensions in vitro. The analyses encompassed platelet activation through assessment of α-granule release and dense-granule exocytosis, in vitro thrombus formation, surface expression of key glycoproteins, activation of integrin α_IIb_β_3_, and evaluation of platelet apoptosis and viability. In a complementary ex vivo component, we examined the effects of asciminib on soluble inflammatory cytokines, platelet–endothelial activation biomarkers, and thrombin generation in platelet-poor plasma (PPP) samples collected from CML patients at baseline and after two months of continuous asciminib treatment.

## 2. Result

### 2.1. Asciminib and Nilotinib Selectively Inhibited G Protein-Coupled Platelet α-Granule Release, Whereas All TKIs Suppressed GPVI/FcRγ-Chain–ITAM-Mediated Platelets α-Granule Release in a Concentration-Dependent Manner

**Figure 1:**
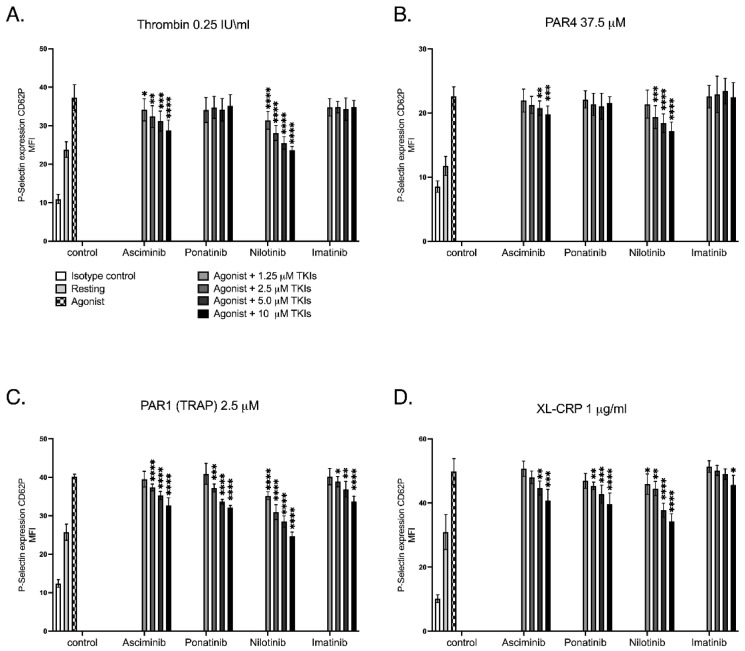
The effects of TKIs on α-granule release in response to different platelet agonists. Washed human platelets (100 × 10^9^/L; 50 µL) were pre-incubated with increasing concentrations (1.25–10 µM) of imatinib, nilotinib, ponatinib, or asciminib for 20 min at 37 °C, followed by stimulation with either (**A**) 0.25 IU/mL thrombin, (**B**) 37.5 µM PAR4, (**C**) 2.5 µM PAR1 (TRAP), or (**D**) 2 µg/mL XL-CRP for 30 min at 37 °C. P-selectin surface expression was quantified by flow cytometry using FITC-conjugated anti-CD62P monoclonal antibody. Each concentration was analysed relative to its corresponding agonist control, and statistical comparisons were performed within each TKI rather than between different TKIs or across matched concentrations. Data are presented as MFI ± SEM from four independent experiments (* *p* < 0.05, ** *p* < 0.01, *** *p* < 0.005, **** *p* < 0.001; *n* = 4; unpaired Student’s *t*-test). Results should be interpreted cautiously given the exploratory nature and limited sample size.

Alpha-granule release represents platelet degranulation, a process that precedes and amplifies platelet activation, and is quantified by flow cytometry through detection of surface P-selectin (CD62P) expression using fluorophore-conjugated antibodies. In this investigation, distinct pathway-dependent effects of BCR::ABL1 tyrosine kinase inhibitors (TKIs) on platelet α-granule release were identified, as shown in Figure 1. Asciminib and Nilotinib markedly reduced α-granule secretion elicited by G-protein-coupled receptor agonists, including thrombin and PAR4, in a concentration-dependent manner. In contrast, Imatinib and Ponatinib produced no appreciable effect under these conditions. When platelets were stimulated through the PAR1(TRAP) receptor, asciminib and ATP pocket inhibitors exhibited a notable inhibitory action. Activation through the collagen receptor GPVI/FcRγ-chain ITAM pathway using CRP-XL, however, revealed a distinct pattern, where all TKIs significantly decreased α-granule release in a dose-dependent manner. Collectively, these data demonstrate that asciminib and nilotinib selectively suppress G-protein-mediated secretory mechanisms while all TKIs converge to dampen GPVI-dependent platelet responses. Such pathway-selective modulation of platelet secretion is consistent with differential regulation of GPCR- and ITAM-dependent signalling cascades in platelets and has been reported for other kinase-targeting agents.

### 2.2. Asciminib Showed No Modulation of Dense-Granule Release in Response to G Protein-Coupled or GPVI-Mediated Platelet Activation

**Figure 2:**
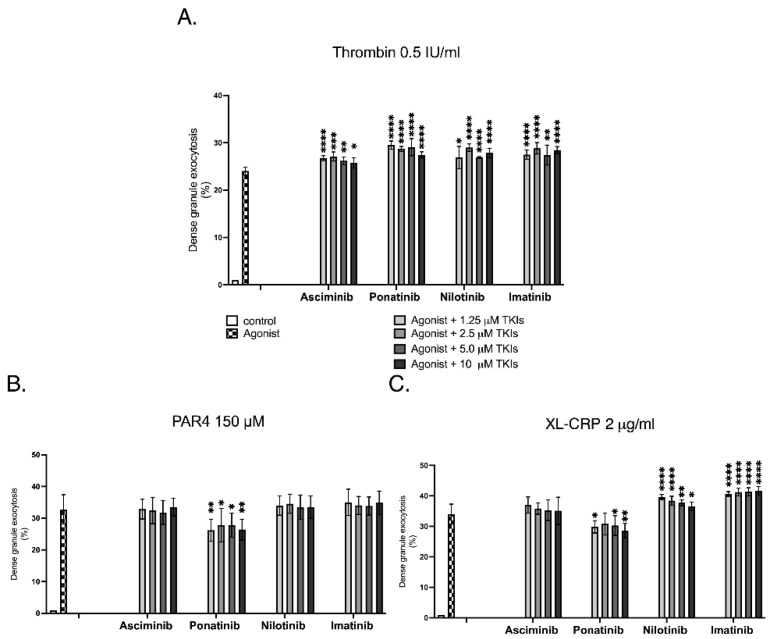
The effect of TKIs on thrombin-mediated platelet dense-granule exocytosis as assessed by flow cytometry. Washed normal human platelets (platelet count normalised to 100 × 10^9^/L) were labelled with 100 µM quinacrine at 37 °C for 1 h in the dark. Labelled platelets were either untreated or pre-incubated with increasing concentrations of asciminib, ponatinib, nilotinib, or imatinib (1.25–10 µM) for 1 h at 37 °C and subsequently stimulated with (**A**) 0.5 IU/mL thrombin, (**B**) 150 µM PAR4, or (**C**) 2 µg/mL XL-CRP for 1 h at 37 °C. Quinacrine-labelled mean fluorescence intensity (MFI) of resting platelets represents dense-granule content, whereas a reduction in MFI following stimulation indicates granule exocytosis. Data are presented as mean ± SEM from four independent experiments (* *p* < 0.05, ** *p* < 0.01, *** *p* < 0.005, **** *p* < 0.001; *n* = 4; unpaired Student’s *t*-test). Results should be interpreted cautiously given the exploratory nature and limited sample size.

Dense-granule exocytosis reflects platelet degranulation that contributes to and sustains platelet activation and was quantified by flow cytometry using quinacrine-labelled platelets, where a decrease in fluorescence intensity indicates release of dense-granule contents such as ADP and serotonin. In this investigation, the effects of BCR::ABL1 tyrosine kinase inhibitors (TKIs) on platelet dense-granule exocytosis were assessed relative to the corresponding agonist-stimulated control for each condition, as illustrated in Figure 2. Asciminib induced a subtle, non-concentration-dependent increase in thrombin-stimulated dense-granule secretion compared with thrombin alone, while showing no modulation following PAR4-AP or CRP-XL activation. Ponatinib similarly induced a subtle, non-concentration-dependent increase in thrombin-stimulated dense-granule secretion relative to agonist control, while producing a non-concentration-dependent reduction in dense-granule exocytosis in response to both PAR4-AP and CRP-XL, suggesting partial interference with G-protein- and GPVI-mediated signalling pathways. Nilotinib enhanced dense-granule secretion following thrombin stimulation relative to agonist control, showed no effect with PAR4-AP, and exhibited a concentration-dependent inhibitory effect in response to CRP-XL activation. Imatinib likewise increased secretion under thrombin and CRP-XL stimulation relative to control but produced no effect following PAR4-AP activation. Collectively, these findings indicate that asciminib exerted minimal effects on platelet dense-granule exocytosis, displaying only a weak, non-concentration-dependent response under thrombin stimulation, whereas ATP-competitive TKIs demonstrated heterogeneous, pathway-specific modulation of secretory activity, underscoring their divergent mechanistic profiles.

### 2.3. Asciminib and Imatinib Reduce In Vitro Thrombus Formation on Type I Collagen in a Concentration-Dependent Manner, Whereas Nilotinib and Ponatinib Enhance Thrombus Formation Under Arterial Flow Conditions

**Figure 3:**
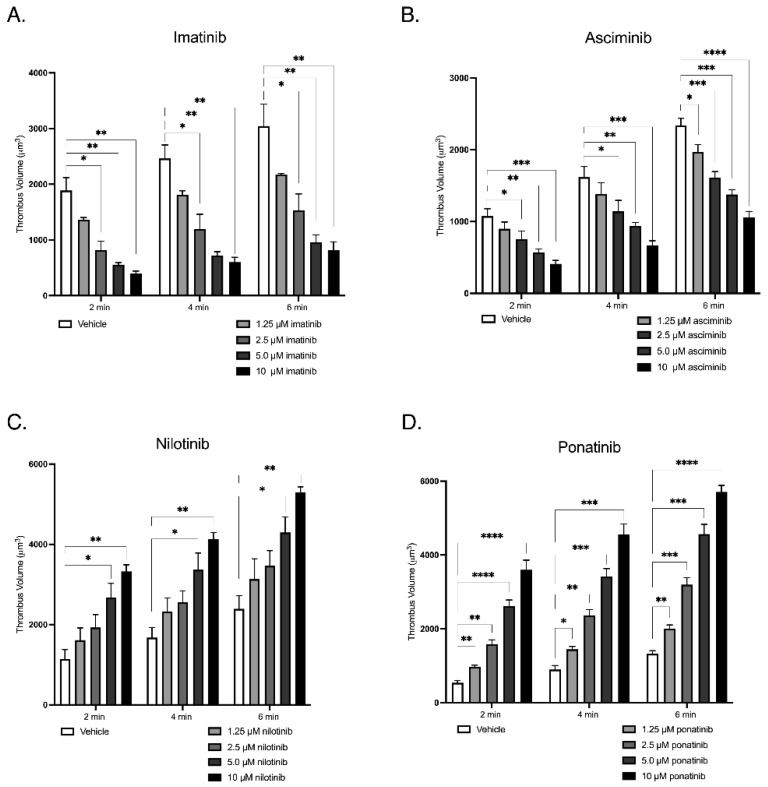
The effects of TKIs on in vitro thrombus formation on type I collagen under arterial shear flow. Fluorescently labelled whole blood was perfused over type I collagen (500 µg/mL) at an arterial shear rate of 1800 s^−1^ for 6 min following pre-incubation with increasing concentrations (1.25–10 µM) of each TKI. Thrombus formation was quantified using confocal microscopy and three-dimensional image analysis. (**A**) imatinib, (**B**) asciminib, (**C**) nilotinib, and (**D**) ponatinib. Data represents summary results from four independent experiments performed on different days using blood from different donors; within each experiment, all concentrations were analysed relative to the corresponding vehicle control run performed in parallel. Asciminib and imatinib decreased thrombus volume in a concentration-dependent manner, whereas nilotinib and ponatinib increased thrombus formation relative to their respective within-experiment controls under arterial shear conditions. Data are presented as mean ± SEM (* *p* < 0.05, ** *p* < 0.01, *** *p* < 0.005, **** *p* < 0.001; *n* = 4 independent experiments). Results should be interpreted cautiously given the exploratory nature and limited sample size.

In vitro thrombus formation under arterial shear flow represents a physiologically relevant model of platelet behaviour under vascular flow, as demonstrated in Figure 3. Fluorescently labelled whole human blood was perfused over type I collagen (500 µg/mL) at an arterial shear rate of 1800 s^−1^ using a Harvard syringe pump. Samples were pre-treated with increasing concentrations (1.25, 2.5, 5.0, and 10.0 µM) of Asciminib, Imatinib, Nilotinib, or Ponatinib, and imaged in real time by fluorescence microscopy. Serial Z-stack images were acquired to reconstruct three-dimensional thrombi, enabling quantitative assessment of thrombus height (µm), area (µm^2^), and volume (µm^3^), providing an integrated measure of platelet adhesion, aggregation, and thrombus formation under arterial shear conditions. Asciminib and Imatinib significantly reduced thrombus volume in a concentration-dependent manner, indicating inhibition of platelet aggregation and thrombus stability. In contrast, Nilotinib and Ponatinib induced a progressive, concentration-dependent increase in thrombus volume, reflecting enhanced platelet deposition and pro-thrombotic potential under arterial flow. This pattern is consistent with prior clinical and experimental evidence linking Nilotinib and Ponatinib to arterial pathology and arteriopathy, supporting the biological relevance of the observed pro-thrombotic phenotype. Collectively, these findings highlight mechanistically divergent effects of TKIs on platelet-driven thrombogenesis, whereby allosteric inhibition (Asciminib) and Imatinib suppress thrombus formation, whereas ATP-competitive inhibitors Nilotinib and Ponatinib potentiate thrombus development under physiological shear conditions.

### 2.4. BCR::ABL1 Tyrosine Kinase Inhibitors Selectively Decrease Platelet GPIbα and GPIX Expression While Preserving GPV and Integrin α_IIb_β_3_

Platelet surface glycoproteins are critical determinants of platelet adhesion and aggregation, mediating the initial capture of circulating platelets to exposed subendothelial collagen and their subsequent interaction with one another. Their expression levels on the platelet surface can be quantitatively measured by flow cytometry, using fluorophore-conjugated monoclonal antibodies specific to each glycoprotein, with results expressed as mean fluorescence intensity (MFI). In this investigation, asciminib, ponatinib, nilotinib, and imatinib each produced a statistically significant reduction in components of the GPIb–IX complex, as shown in Table 1. GPIbα expression was consistently decreased (*p* < 0.0001), while GPIX expression was also significantly reduced, reaching *p* < 0.005 in asciminib- and ponatinib-treated platelets. In contrast, GPV and surface integrin α_IIb_β_3_ expression were not significantly altered (*p* > 0.05). Collectively, these data indicate selective modulation of the GPIb–IX adhesion axis by BCR::ABL1 TKIs.

### 2.5. BCR::ABL1 TKIs Suppress Integrin α_IIb_β_3_ Activation, Induce Platelet Apoptosis with Asciminib and Ponatinib, and Show No Effect on Platelet Viability

**Figure 4:**
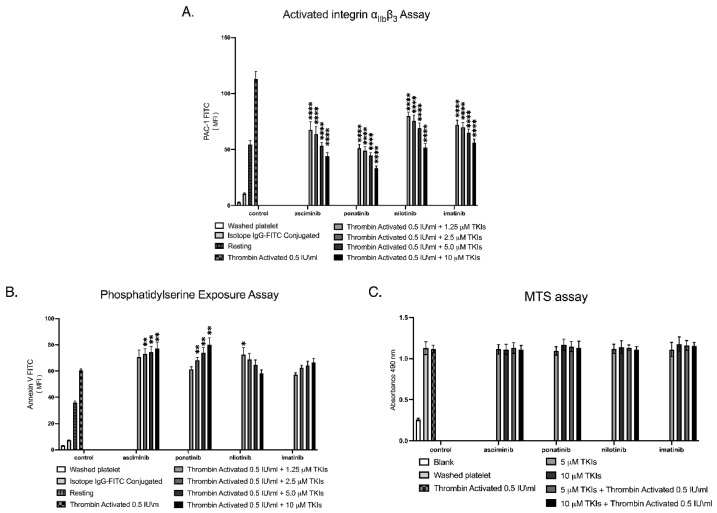
Effects of TKIs on integrin α_IIb_β_3_ activation, platelet apoptosis, and platelet viability. (**A**) Integrin α_IIb_β_3_ activation was quantified by PAC-1 binding following pre-incubation of washed platelets with increasing concentrations of asciminib, ponatinib, nilotinib, or imatinib (1.25–10 µM). All TKIs produced a dose-dependent reduction in PAC-1 binding, indicating suppression of integrin activation. (**B**) Platelet apoptosis was assessed by phosphatidylserine exposure using Annexin V binding. Asciminib and ponatinib induced a dose-dependent increase in Annexin V-positive platelets, whereas nilotinib and imatinib showed minimal or no apoptotic effect. (**C**) Platelet viability was assessed using the MTS assay, measuring mitochondrial metabolic activity with or without 0.5 IU/mL thrombin stimulation. No TKI produced a measurable reduction in viability across all concentrations tested. Data are presented as mean ± SEM from four independent experiments (* *p* < 0.05, ** *p* < 0.01, **** *p* < 0.001; *n* = 4; unpaired Student’s *t*-test). Results should be interpreted cautiously given the exploratory nature and limited sample size.

Activated integrin α_IIb_β_3_ represents the conformationally active form of the fibrinogen receptor that enables platelet aggregation, and its surface activation state can be quantified by flow cytometry using the PAC-1 monoclonal antibody, which selectively binds the ligand-competent (active) conformation of α_IIb_β_3_. In this investigation, the effects of TKIs on activated integrin α_IIb_β_3_ were assessed using the PAC-1 binding assay, as shown in Figure 4A. All TKIs produced a sharp, dose-dependent reduction in PAC-1 binding, indicating strong suppression of integrin activation and impaired platelet aggregation capacity.

Platelet apoptosis was assessed by measuring phosphatidylserine exposure on the outer membrane leaflet, using an Annexin V-binding assay quantified by flow cytometry, which detects early apoptotic changes with high specificity. In this investigation, platelet apoptosis was assessed by Annexin V binding to phosphatidylserine-expressing platelets, as shown in Figure 4B. Asciminib and Ponatinib induced a clear, dose-dependent increase in Annexin V-positive platelets, whereas Imatinib and Nilotinib showed minimal or no apoptotic effect across the concentrations tested.

Platelet viability was assessed using the MTS assay, which quantifies mitochondrial metabolic activity as a surrogate marker of platelet health and lifespan, with absorbance measurements reflecting the proportion of metabolically active, surviving platelets following TKI exposure in the presence or absence of 0.5 IU/mL thrombin stimulation. In this investigation, platelet viability was examined following TKI exposure using the MTS assay, as shown in Figure 4C. Across all concentrations tested, and both in the presence and absence of thrombin stimulation, none of the TKIs produced a measurable reduction in platelet viability, indicating preserved metabolic activity and platelet lifespan under all treatment conditions. These findings are based on a small sample size (*n* = 4) and should be interpreted with caution.

### 2.6. Selective Inhibition of Soluble Inflammatory Cytokines and Soluble Platelet–Endothelial Biomarkers by Asciminib in Ex Vivo-Treated CML Patients

**Figure 5:**
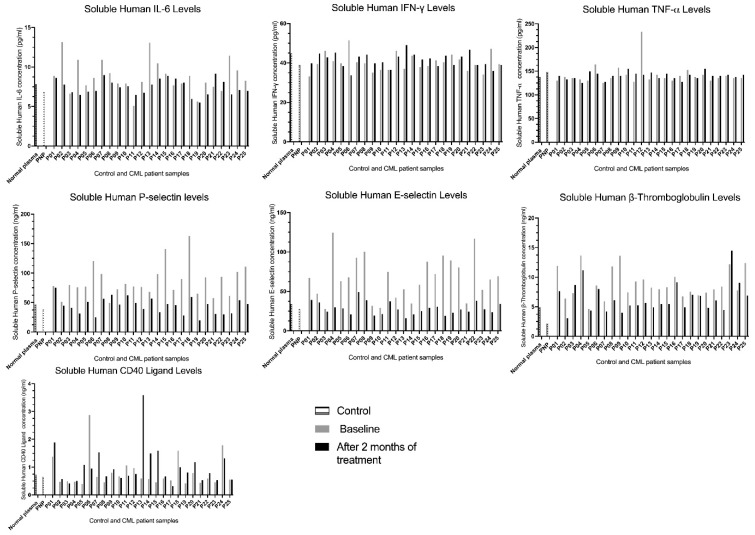
Soluble inflammatory cytokine and platelet endothelial biomarker profiles in CML patients at baseline and after two months of asciminib therapy. Plasma concentrations of IL-6, IFN-γ, and TNF-α were measured in paired samples from 25 chronic-phase CML patients using sandwich ELISA. Each bar set represents an individual patient, with baseline and two-month asciminib treatment values plotted side-by-side. IL-6 exhibited heterogeneous responses, with both increases and decreases observed following treatment. IFN-γ and TNF-α showed minimal or no consistent modulation across patients. Data illustrates substantial inter-individual variability in cytokine responses during asciminib therapy. No formal statistical comparisons were performed.

Soluble inflammatory cytokines assessed in this investigation included IL-6, which exhibits both pro- and anti-inflammatory activity, together with the strictly pro-inflammatory mediators IFN-γ and TNF-α that signal immune activation. Soluble platelet–endothelial biomarkers comprised P-selectin released from platelet α-granules and endothelial Weibel–Palade bodies E-selectin as an endothelial activation marker, β-thromboglobulin derived from platelet α-granules, and CD40L expressed by activated platelets and T cells. These molecules appear in plasma in their soluble form through proteolytic shedding from the cell surface or granule exocytosis, providing measurable indicators of platelet and endothelial activation. In this investigation, we examined the effects of asciminib on soluble inflammatory cytokines and soluble platelet–endothelial biomarkers in a cohort of 25 chronic-phase CML patients, assessed at baseline and again after two months of continuous asciminib treatment, using a sandwich ELISA approach. The cohort included 12 females and 13 males, with ages ranging from 23 to 92 years; however, neither sex nor age influenced biomarker responses in this study. As shown in Figure 5, individual patient responses displayed substantial variability, with some patients exhibiting marked reductions and others showing sharp increases across different analytes, and this heterogeneity varied from one biomarker to another. These paired bar graphs therefore represent the individual biological trajectories of each patient, highlighting that asciminib ’s modulatory effects are not uniform but patient-specific across the soluble cytokine and endothelial–platelet biomarker panels. Baseline vascular comorbidities and concomitant medications are provided in Supplementary Table S1.

As shown in Figure 5, asciminib was associated with distinct biomarker-specific inflammatory responses across the CML cohort. sIL-6 showed predominant suppression (76% inhibition, 24% potentiation), whereas sIFN-γ and sTNF-α exhibited heterogeneous but largely increased responses, with 60% potentiation for each and 32–36% inhibition, reflecting inter-individual variability in baseline inflammatory and immune status. In contrast, platelet–endothelial activation markers were generally reduced, with sP-selectin inhibited in 96% of patients, sE-selectin in 100%, and sβ-thromboglobulin in 88%, suggesting a trend toward attenuation of platelet and endothelial activation. sCD40L displayed a potentiation-dominant profile (64% potentiation), consistent with its role in immune and inflammatory signalling. Collectively, these findings suggest that asciminib may be associated with reduced platelet–endothelial activation, while immune-associated cytokines exhibit patient-dependent modulation. These observations are descriptive and should be interpreted with caution given the absence of a control group and potential confounding variables. No formal paired statistical analyses were performed for these observations.

### 2.7. Asciminib Is Associated with Increased Thrombin Generation in Ex Vivo Plasma from CML Patients

**Figure 6:**
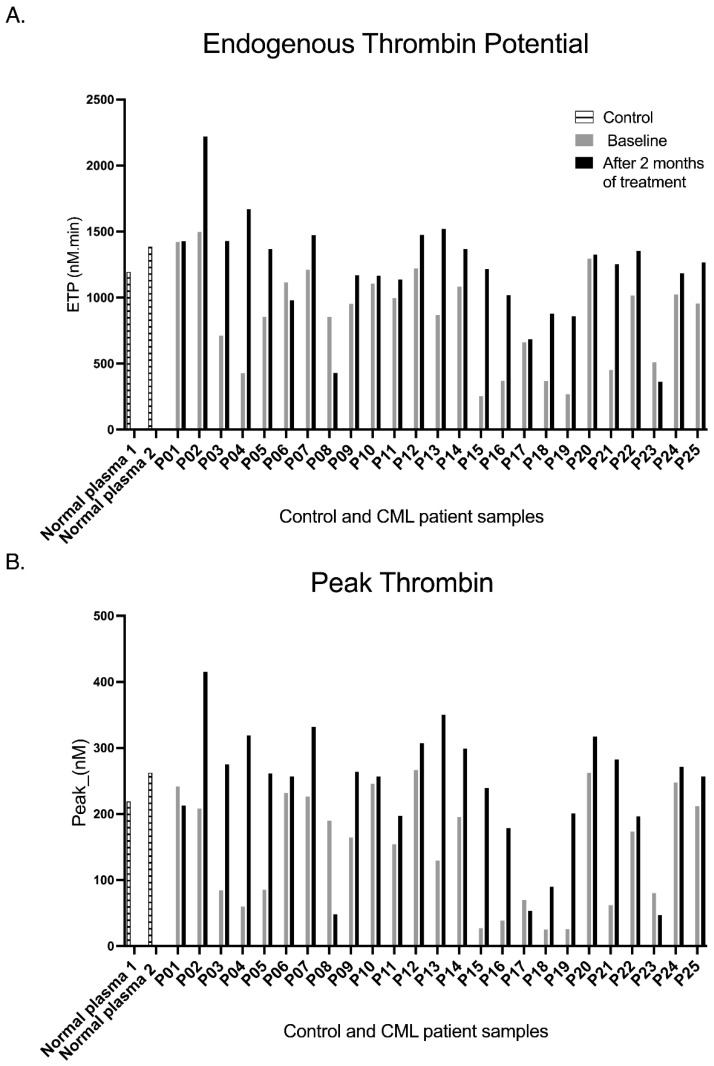
Individual effects of asciminib on thrombin generation in CML patients ex vivo. (**A**) Endogenous Thrombin Potential; (**B**) Peak Thrombin. Baseline and two-month paired plasma samples from 25 chronic-phase CML patients were analysed using calibrated automated thrombography (CAT) to quantify endogenous thrombin potential (ETP) and peak thrombin. Individual bar plots demonstrate increased thrombin generation in the majority of patients following asciminib treatment, with potentiation of both ETP and peak thrombin observed in most cases. Data are presented descriptively, and no formal statistical comparisons were performed.

Thrombin generation provides an integrated measure of coagulation activity and hypercoagulability, with elevations in Endogenous Thrombin Potential (ETP) and Peak Thrombin strongly associated with venous and arterial thrombosis across diverse clinical settings. In this investigation, calibrated automated thrombography was applied to paired plasma samples, and as shown in Figure 6, asciminib was associated with increased thrombin generation at the individual-patient level, with most patients demonstrating clear potentiation of both ETP and Peak Thrombin. No formal paired statistical analyses were performed for these observations.

Asciminib was associated with increased thrombin generation across the CML cohort. Endogenous Thrombin Potential (ETP) was increased in 88% of patients (with 12% showing inhibition), indicating an upward shift in global thrombin-generating capacity. Notably, several patients exhibited relatively low baseline thrombin generation, such that normalisation to individual baseline values may further accentuate the magnitude of ETP increase in this subgroup. Peak Thrombin showed a similar pattern, with 84% of patients exhibiting increases and 16% inhibition, suggesting enhanced thrombin burst amplitude following treatment. Collectively, these findings suggest an increase in thrombin generation in most patients. These observations are descriptive and should be interpreted with caution given the absence of a control group and potential confounding variables.

## 3. Discussion

Tyrosine kinase inhibitors (TKIs) have transformed the management of chronic myeloid leukaemia (CML), markedly reducing mortality following the introduction of imatinib [11]. However, resistance and intolerance led to the development of second- and third-generation ATP-competitive TKIs, including nilotinib and ponatinib [12,13]. Despite improved mutation coverage, both agents are associated with clinically significant vascular toxicity, including arterial occlusive and thromboembolic events [6,7,14]. These observations have raised concerns regarding TKI-associated prothrombotic liability mediated through platelet activation and endothelial dysfunction.

Asciminib, a first-in-class STAMP inhibitor targeting the ABL1 myristoyl-binding site, was developed to improve selectivity and safety relative to ATP-competitive TKIs [1,2,3,10]. The present study suggests that asciminib exhibits a haemostatic profile distinct from ATP-binding TKIs. Across in vitro platelet assays, arterial shear flow thrombus models, and plasma analyses from asciminib-treated CML patients, asciminib consistently attenuated primary haemostasis. Platelet activation, adhesion receptor expression (GPIbα, GPIX), integrin α_IIb_β_3_ activation, and thrombus formation on type I collagen were significantly reduced. Asciminib induced dose-dependent phosphatidylserine externalisation, indicating activation of platelet apoptotic signalling. However, platelet metabolic activity assessed by MTS assay remained preserved, suggesting modulation of apoptotic pathways without overt cytotoxicity. These findings are based on a small sample size (*n* = 4) and should be interpreted with caution. Although platelet viability remained preserved, increased phosphatidylserine exposure may contribute to a procoagulant surface under certain conditions, and its clinical significance warrants further investigation.

These findings contrast with prior reports demonstrating that nilotinib and ponatinib enhance platelet activation and thrombus formation under arterial shear [6,7]. Clinical and experimental data have linked these ATP-competitive TKIs to increased thrombotic risk [15]. The attenuation of platelet-driven arterial thrombus formation observed with asciminib may suggest a comparatively less prothrombotic profile in vitro.

The concentrations used in vitro (up to 10 µM) exceed typical steady-state plasma levels reported clinically and do not account for protein binding or pharmacokinetic constraints that influence free drug availability in vivo. Furthermore, the use of washed platelet systems excludes plasma components essential for haemostatic regulation, limiting physiological relevance. These findings should therefore be interpreted within the context of simplified experimental conditions.

Exploratory cytokine analyses revealed a reduction in IL-6 following asciminib treatment, whereas TNF-α and IFN-γ changes were heterogeneous and non-uniform. Given their variability and modest magnitude, these cytokine alterations are more plausibly attributed to underlying disease activity rather than direct drug effects. In contrast, platelet-derived biomarkers, particularly soluble P-selectin and soluble E-selectin, showed consistent reductions across the cohort, reinforcing a platelet-centred mechanism of action.

Notably, sCD40L increased in a subset of patients and paralleled TNF-α elevation. CD40L is expressed by both platelets and immune cells and participates in inflammatory amplification loops [14,16]. Baseline sCD40L levels were low in several patients prior to treatment, suggesting that subsequent increases are likely to reflect residual immune activation rather than a direct prothrombotic drug effect. This interpretation is supported by the absence of concurrent increases in platelet activation markers.

Concomitant antiplatelet and anticoagulant medications did not demonstrate a consistent association with changes in soluble biomarkers. Observed responses were heterogeneous and did not correlate with background vascular therapy, suggesting that the haemostatic alterations were more likely attributable to asciminib rather than concomitant medication use. In addition, asciminib-treated CML patients achieved >90% haematological and cytogenetic remission after the 2-month treatment.

Despite suppression of primary haemostasis, asciminib treatment was associated with increased thrombin generation, reflected by elevations in endogenous thrombin potential and peak thrombin [17,18]. Thrombin generation assays primarily reflect plasma coagulation factor dynamics rather than direct platelet activation. This apparent dissociation may reflect biological separation between platelet-driven primary haemostasis and plasma-driven secondary haemostasis and may indicate a true shift in plasma procoagulant potential, a compensatory response to reduced platelet activation, or a methodological feature of the assay performed in platelet-poor plasma rather than whole blood. This observation may reflect differential modulation of coagulation factor activity or plasma procoagulant balance independent of platelet activation, potentially involving tissue factor-driven pathways or alterations in anticoagulant regulators. However, no direct assessment of coagulation factors, endothelial contribution, or pathway-specific mechanisms was performed in this study. Similar thrombin-enhancing effects have been reported with nilotinib [6], suggesting that modulation of secondary haemostasis may represent a broader TKI-associated phenomenon. Notably, thrombin generation parameters did not demonstrate a consistent association with concomitant anticoagulant therapy, although this observation should be interpreted cautiously given the limited sample size. While these findings do not establish clinical thrombotic risk, they highlight that the procoagulant potential of asciminib, particularly in patients with pre-existing vascular risk factors or advanced age, warrants careful clinical consideration.

Increased thrombin generation has been associated with venous thrombosis and ischaemic stroke risk in other clinical settings [19]. However, the present study did not assess clinical thrombotic outcomes, longitudinal vascular events, or validated surrogate endpoints, and therefore these findings should not be interpreted as evidence that asciminib increases thrombotic risk. The increase observed with asciminib may instead reflect indirect modulation of coagulation factor balance or anticoagulant regulation rather than endothelial injury or platelet hyperreactivity. This mechanistic distinction may differentiate asciminib from the endothelial-disruptive effects reported for ponatinib and nilotinib [20,21].

No formal correlation analyses were performed between thrombin generation parameters and soluble platelet–endothelial or inflammatory biomarkers. Given the inter-individual variability and exploratory nature of the dataset, such analyses would require larger cohorts to provide meaningful inference. Future studies integrating paired biomarker and coagulation analyses may help further define the relationship between platelet-driven and plasma-driven haemostatic responses.

The present findings are primarily phenomenological and do not include direct interrogation of intracellular signalling pathways. However, asciminib, as an allosteric inhibitor of BCR::ABL1, may influence downstream signalling cascades relevant to platelet function and coagulation, including pathways linked to Src family kinases, PI3K/AKT, and MAPK signalling. Modulation of these pathways may contribute to the observed effects on platelet activation and thrombin generation. Nevertheless, no direct mechanistic or pathway-specific analyses were performed in this study, and these interpretations remain speculative.

Preclinical studies further suggest a comparatively differentiated vascular profile of asciminib. Ponatinib and nilotinib impair endothelial repair and promote thrombosis in murine models, whereas asciminib does not demonstrate these effects [20,21]. Clinical analyses similarly report lower rates of arterial occlusive events with asciminib compared with ATP-competitive TKIs [15]. Collectively, these data are consistent with the present mechanistic findings, although they do not establish clinical vascular safety in the current cohort.

**Figure 7:**
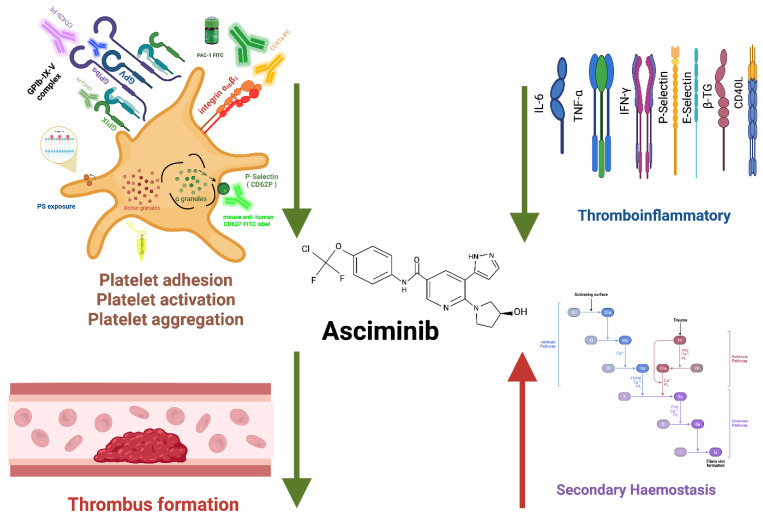
Summary of the effects of asciminib on platelet primary haemostasis, thrombus formation, thromboinflammatory state and coagulation cascade. This diagram illustrates the multilevel effect of asciminib, an allosteric myristoyl pocket inhibitor of BCR::ABL1, on platelet activation pathways and haemostatic balance. Asciminib inhibits primary haemostasis functions, including platelet adhesion (via decreased expression of GPIbα and GPIX), platelet activation (via decreased α granule release through P-selectin expression), and platelet aggregation (by attenuating the activation of integrin α_IIb_β_3_). Asciminib may be associated with reduced in vitro thrombus formation of type I collagen under arterial shear flow. Asciminib also exerts anti-thromboinflammatory effects by decreasing the levels of inflammatory cytokine and platelet/endothelium biomarkers, such as soluble interleukin-6, sP-selectin, sE-selectin, and beta-thromboglobulin. All inhibition effects by asciminib are indicated by the downward-pointing green arrows. In contrast, asciminib enhanced thrombin generation by modifying secondary haemostasis, as illustrated by the red arrow pointing upward toward the coagulation pathway. This figure summarises the dual role of asciminib in inhibiting platelet-mediated inflammation while potentially enhancing procoagulant activity, highlighting its complex vascular effects in chronic phase CML patient treatment.

The study has several limitations. Whole blood samples from asciminib-treated patients were not available for direct ex vivo thrombus formation analysis, the cohort size was limited to 25 patients, and the follow-up duration was restricted to two months. The use of platelet-poor plasma for thrombin generation analysis may also limit direct comparison with in vitro platelet function and thrombus formation assays. Nevertheless, consistent biomarker trends and dose-dependent in vitro effects across assays support the robustness of the findings. A schematic summary of the proposed haemostatic effects of asciminib is illustrated in Figure 7.

## 4. Materials and Methods

### 4.1. Drugs

Asciminib (ABL001), imatinib, nilotinib, and ponatinib were purchased from Selleck Chemicals (Houston, TX, USA). Stock solutions (10 mM) were prepared in dimethyl sulfoxide (DMSO) (Melbourne, Victoria), diluted in Tyrode’s buffer as required, and stored at −80 °C. The final DMSO concentration in all experiments was <0.2% (*v*/*v*).

Working concentrations (1.25–10 μM) were selected to encompass clinically relevant plasma exposures and supra-physiological ranges commonly used in vitro to assess concentration-dependent target inhibition under protein-containing conditions. These concentrations exceed typical free plasma levels observed in patients but are consistent with in vitro experimental conditions where protein binding, drug distribution, and target engagement differ from the in vivo setting.

### 4.2. Study Participants and Ethics

The study was approved by the Central Adelaide Local Health Network Human Research Ethics Committee (CALHN HREC; approval reference R20200803) and the RMIT University Human Research Ethics Committee (approval number 28450). All participants provided written informed consent in accordance with the Declaration of Helsinki.

### 4.3. Platelet Analysis and Preparation

Haematological parameters were determined in platelet-rich plasma or whole blood as previously described [6]. Human platelets were washed and prepared [22] and used immediately for flow cytometric analysis. Data acquisition was performed on a BD FACSCanto II cytometer (Becton Dickinson, North Ryde, Sydney, NSW, Australia), with 10,000 platelet events gated by forward and side scatter. Results were analysed using Weasel software (Version 3.8.2) and expressed as mean fluorescence intensity.

### 4.4. Platelet Functional Assays

α-granule release was assessed by surface P-selectin (CD62P) expression using flow cytometry. Washed platelets (100 × 10^9^/L) were pre-incubated with TKIs (1.25–10 μM) or vehicle (15 min, room temperature), stimulated with thrombin, PAR1, PAR4, or XL-CRP, and analysed by FITC–anti-CD62P staining, with mean fluorescence intensity as the readout. Dense granule secretion was evaluated by quinacrine release following TKI incubation (1.25–10 μM, 60 min) and agonist stimulation, with loss of fluorescence quantified by flow cytometry and expressed as percentage release.

### 4.5. In Vitro Thrombus Formation Under Arterial Shear on Type I Collagen

Thrombus formation was assessed in rhodamine 6G-labelled whole blood perfused over type I collagen-coated microfluidic channels (Ibidi μ-slide VI 0.1) at arterial shear (1800 s^−1^). Blood was pre-incubated with TKIs (1.25–10 μM, 30 min, 37 °C), perfused for 2–6 min, and imaged by fluorescence microscopy. Thrombus area, height, and volume were quantified from z-stack reconstructions.

### 4.6. Platelet Surface Receptors and Integrin Activation

Surface expression of GPIbα, GPIX, GPV, and integrin αIIbβ3 was assessed by flow cytometry in washed platelets pre-incubated with TKIs (1.25–10 μM, 60 min, 37 °C). Integrin activation was quantified by PAC-1 binding following thrombin stimulation (0.5 U/mL), with mean fluorescence intensity used as the readout.

### 4.7. Platelet Apoptosis and Viability

Phosphatidylserine externalisation was assessed by Annexin V binding following TKI treatment and thrombin stimulation, with mean fluorescence intensity used as an index of apoptosis. Platelet metabolic activity was measured using the MTS assay after exposure to TKIs (5 or 10 μM), with or without thrombin.

### 4.8. Soluble Biomarkers and Thrombin Generation

Plasma cytokines (IL-6, IFN-γ, TNF-α) and platelet–endothelial biomarkers (sP-selectin, sE-selectin, sβ-thromboglobulin, sCD40L) were quantified in platelet-poor plasma by sandwich ELISA according to the manufacturers’ protocols. Thrombin generation was assessed using calibrated automated thrombography, with lag time, peak thrombin, time to peak, and endogenous thrombin potential recorded. Baseline vascular comorbidities and concomitant medications are summarised in Supplementary Table S1.

### 4.9. Statistical Analysis

Data are presented as mean ± SEM. Statistical analyses were performed using GraphPad Prism 9.4. Unpaired Student’s *t*-test or one-way ANOVA with appropriate post hoc testing was applied where indicated, with *p* ≤ 0.05 considered statistically significant. Due to the exploratory nature and small sample size of the in vitro experiments (*n* = 4), statistical analyses are limited and should be interpreted with caution. For ex vivo data, although repeated measures were available, formal paired statistical analyses were not consistently applied, and results are therefore presented primarily descriptively, limiting longitudinal inference.

## 5. Conclusions

Asciminib may be associated with a haemostatic profile distinct from ATP-competitive BCR::ABL1 inhibitors. The drug may attenuate primary haemostatic processes and platelet–endothelial activation while preserving platelet viability. Concurrent enhancement of thrombin generation may indicate modulation of secondary haemostasis, suggesting a mechanistic dissociation between platelet-driven and plasma-driven pathways. Compared with nilotinib and ponatinib, asciminib may exhibit a comparatively less prothrombotic mechanistic profile in vitro and ex vivo, although clinical thrombotic risk cannot be inferred from the present study. These findings provide mechanistic insight into asciminib’s vascular profile and support further investigation of its long-term thrombotic implications in CML, particularly in high-risk or elderly patient populations.

These findings should be interpreted with caution given the observational nature of the ex vivo dataset, the absence of a control group, potential confounding variables, substantial inter-individual variability in biomarker responses, reliance on descriptive rather than inferential statistical analysis, and the lack of clinical outcome or longitudinal follow-up data.

## Figures and Tables

**Table 1:** Effects of Tyrosine Kinase Inhibitors (5 µM) on Platelet Surface Glycoprotein Expression in Resting Human Platelets.

TKI/GPs	GPIbα MFI Resting	GPIbα MFI 5 μM	GPIbα *p*-Value	GPIX MFI Resting	GPIX MFI 5 μM	GPIX *p*-Value	GPV MFI Resting	GPV MFI 5 μM	GPV *p*-Value	Integrin α_IIb_β_3_ MFI Resting	Integrin α_IIb_β_3_ MFI 5 μM	Integrin α_IIb_β_3_ *p*-Value
Asciminib	160.6	140.1	*p* < 0.0001	629.2	583.7	*p* < 0.005	46.9	46.5	*p* > 0.05	106.4	104.8	*p* > 0.05
Ponatinib	160.6	143.3	*p* < 0.0001	629.2	584.4	*p* < 0.005	46.9	45.4	*p* > 0.05	106.4	106.4	*p* > 0.05
Nilotinib	160.6	130.9	*p* < 0.0001	629.2	546.6	*p* < 0.0001	46.9	44.6	*p* > 0.05	106.4	104.2	*p* > 0.05
Imatinib	160.6	126.2	*p* < 0.0001	629.2	564.0	*p* < 0.0001	46.9	44.0	*p* > 0.05	106.4	106.4	*p* > 0.05

## Data Availability

The data presented in this study are available on request from the corresponding author.

## Supplementary Materials

The following supporting information can be downloaded at: https://www.mdpi.com/article/10.3390/ijms27083623/s1.
