# Supplementary material for: Allosteric and ATP-Pocket BCR::ABL1 Inhibition In Vitro, and Characterising Ex Vivo Thrombo-Inflammatory Biomarkers and Thrombin Generation in Asciminib-Treated CML Patients
Source: Int J Mol Sci. 2026 Apr 18;27(8):3623. doi: 10.3390/ijms27083623 (PMC13116958; doi:10.3390/ijms27083623)
Supplement: Supplementary file 1 [file ijms-27-03623-s001.zip › ijms-4203155-supplementary.pdf]

International Journal of *Molecular Sciences*

MDPI

## Supplementary Table S1

Baseline vascular comorbidities and concomitant medications in asciminib-treated CML patients.

| Patient No. | Vascular comorbidities / risk factors | Vascular events reported | Concomitant vascular medications (classified) |
|---|---|---|---|
| P1 | Dyslipidaemia | None recorded | rosuvastatin; metoprolol |
| P2 | Data not available | None recorded | Data not available |
| P3 | Ischaemic heart disease | None recorded | enoxaparin |
| P4 | Data not available | None recorded | aspirin |
| P5 | Data not available | None recorded | Data not available |
| P6 | Dyslipidaemia; hypertension; ischaemic heart disease | None recorded | clopidogrel; rosuvastatin; ezetimibe; amlodipine, perindopril |
| P7 | Peripheral arterial occlusive disease; prior cerebrovascular accident; hypertension; femoro-popliteal disease; prior coronary artery bypass grafting | Peripheral arterial occlusive disease | aspirin, ticagrelor; atorvastatin; amlodipine, candesartan, hydrochlorothiazide, metoprolol, perindopril; empagliflozin, insulin degludec/aspart, metformin |
| P8 | Dyslipidaemia; ischaemic heart disease | None recorded | rosuvastatin; metoprolol; empagliflozin |
| P9 | Hypertension; dyslipidaemia; diabetes mellitus with neuropathy | Stroke; transient ischaemic attack | aspirin, clopidogrel; apixaban; rosuvastatin; metoprolol tartrate; empagliflozin, gliclazide, metformin |
| P10 | Ischaemic heart disease | None recorded | Data not available |
| P11 | Dyslipidaemia; hypertension; ischaemic heart disease | None recorded | rosuvastatin; atenolol, indapamide, telmisartan |
| P12 | Hypertension; ischaemic heart disease | None recorded | amlodipine, indapamide, ramipril |
| P13 | Ischaemic heart disease | None recorded | Data not available |
| P14 | Data not available | None recorded | Data not available |
| P15 | Ischaemic heart disease | None recorded | Data not available |
| P16 | Cerebrovascular disease; ischaemic heart disease | None recorded | Data not available |
| P17 | Hypertension; dyslipidaemia; aortic stenosis | Chest pain; LAD disease on angiography | aspirin, clopidogrel; heparin; simvastatin; bisoprolol, hydrochlorothiazide, metoprolol, ramipril, spironolactone |

| | | | |
|---|---|---|---|
| P18 | Hypertension; dyslipidaemia; ischaemic heart disease | Internal carotid artery stenosis; diffuse peripheral vascular disease (ultrasound) | clopidogrel; pravastatin; antihypertensive: perindopril |
| P19 | Dyslipidaemia; hypertension; ischaemic heart disease | None recorded | rosuvastatin; amlodipine, perindopril, spironolactone |
| P20 | Hypertension; dyslipidaemia; diabetes mellitus with nephropathy | End-stage renal failure | aspirin; enoxaparin, heparin; atorvastatin, simvastatin; ezetimibe; antihypertensive: amlodipine, metoprolol, perindopril; empagliflozin, linagliptin, metformin |
| P21 | Ischaemic heart disease | None recorded | Data not available |
| P22 | Atrial fibrillation; diabetes mellitus; dyslipidaemia; hypertension; ischaemic heart disease | None recorded | aspirin; atorvastatin; dapagliflozin, metformin |
| P23 | Ischaemic heart disease | None recorded | Data not available |
| P24 | Dyslipidaemia; ischaemic heart disease | None recorded | Data not available |

*Notes: Patient identifiers are anonymised and presented as sequential numbers. Vascular comorbidities, events, and concomitant medications are reported only where documented in the source clinical dataset; absence of information indicates unavailable data rather than absence of disease. Medication classes are summarised for contextual purposes and were not used for stratified statistical inference.*
